# The Evaluation of SWEEPS Plus Antimicrobial Photodynamic Therapy with Indocyanine Green in Eliminating *Enterococcus faecalis* Biofilm from Infected Root Canals: An In Vitro Study

**DOI:** 10.3390/biomedicines11071850

**Published:** 2023-06-28

**Authors:** Golriz Rostami, Shima Afrasiabi, Stefano Benedicenti, Antonio Signore, Nasim Chiniforush

**Affiliations:** 1 Laser Research Center of Dentistry, Dentistry Research Institute, Tehran University of Medical Sciences, Tehran 1441987566, Iran; golriz.rostami@gmail.com (G.R.); shafrasiabi@alumnus.tums.ac.ir (S.A.); 2 Department of Surgical Sciences and Integrated Diagnostics, University of Genoa, Viale Benedetto XV 6, 16132 Genoa, Italy; benedicenti@unige.it; 3 Therapeutic Dentistry Department, Institute of Dentistry, I.M. Sechenov First Moscow State Medical University, Trubetskaya Str., 8, b. 2, 119992 Moscow, Russia; dr.signore@icloud.com

**Keywords:** antimicrobial photodynamic therapy, biofilms, disinfection, Er:YAG laser, *Enterococcus faecalis*, indocyanine green, hypochlorite sodium, SWEEPS

## Abstract

Objectives: This study aimed to assess the efficacy of shockwave-enhanced emission photoacoustic streaming (SWEEPS) plus antimicrobial photodynamic therapy (aPDT) using indocyanine green (ICG) for the elimination of *Enterococcus faecalis* biofilm from infected root canals. Materials and Methods: thirty sound human single-canal teeth were chosen and standardized to have 12 mm of root length. The root canals were shaped and prepared by means of ProTaper rotary files. After sterilization of the teeth, the canals were inoculated with *E. faecalis* for 2 weeks. The teeth were then randomly divided into six groups (n = five) of control, ICG, ICG + 808 nm diode laser, ICG + SWEEPS, ICG + 808 nm diode laser + SWEEPS, and 5.25% sodium hypochlorite (NaOCl). Following treatment, the number of colony-forming units (CFUs)/mL were calculated for each group. Statistical analysis was carried out using one-way ANOVA. For multiple comparisons, Tukey’s test was used as the post hoc test. Results: NaOCl alone showed the highest efficacy (*p* < 0.001). The ICG + 808 nm diode laser + SWEEPS group displayed significantly lower amounts of bacteria than either the ICG + 808 nm diode laser or SWEEPS (*p* < 0.001). There was a statistically significant difference detected between the ICG + 808 nm diode laser and ICG + SWEEPS (*p* = 0.035). Conclusions: SWEEPS can effectively increase the photosensitizer distribution in the root canal space, and its application along with irrigants can bring about promising results.

## 1. Introduction 

Endodontic treatments aim to effectively reduce the microorganisms responsible for endodontic infections [1]. However, the complete elimination of endodontic pathogens is extremely difficult, if not impossible, with the commonly used instrument methods, due to the complex anatomy of the root canal system and the presence of lateral canals, isthmi, ramifications and fins [2]. *Enterococcus faecalis* is associated with secondary endodontic infections, refractory infected lesions and periapical biofilms, resulting in endodontic treatment failure [3]. Teeth with failed endodontic treatment are more likely than non-endodontically treated teeth to contain this microorganism in their root canal system [3]. The resistance of this bacterium to the challenges of survival within the root canal space is related to the ability to invade the dentinal tubules and bond to collagen fibers, biofilm formation, and its capacity to endure harsh environments [3]. 

Root canal irrigation is performed along with mechanical cleaning and instrumentation of canals to chemically decrease the intracanal microbial load. Syringe irrigation is the standard method of root canal irrigation. The elimination of bacterial biofilm is not possible merely by the chemical action of irrigants or mechanical instrumentation alone, and chemical irrigants should be used in combination with physical manipulation of the canal in order to be able to access all parts of the root canal system [4]. Instrumentation with rotary and hand files cannot efficiently clean the isthmi and canal irregularities, and approximately 35% of the canal surface always remains intact [5]. In addition, rotary instruments create significant amounts of dentinal debris that may accumulate in the canal irregularities and isthmi. The presence of debris prevents the optimal sealing of the canal with root filling materials, and can impair efficient root canal disinfection [6]. Sodium hypochlorite (NaOCl) is a root canal irrigating solution that is currently the most popular, since it can remove bacteria and their biofilm and dissolve the residual vital and necrotic tissues [7]. Nevertheless, NaOCl has neurotoxic and cytotoxic effects, and exhibits a destructive effect on mineralized dentin [8]. Different techniques are used to enhance the efficacy and penetration depth of irrigants into the canal irregularities, such as sonic and ultrasonic instruments and different types of lasers [9].

Laser application for the activation of root canal irrigants and elimination of debris accumulated in the canal has gained increasing attention in recent years. In antimicrobial photodynamic therapy (aPDT), the root canals are filled with a light-sensitive material known as photosensitizer, which is then activated with the appropriate wavelength of light, and produces singlet oxygen and other free radicals in the presence of oxygen molecules. Free oxygen radicals damage the microbial molecules such as proteins, membrane lipids and nucleic acid, and cause microbial death [10]. 

Indocyanine green (ICG) (4,5-benzoindotricarbocyanine—C43H47N2NaO6S2), also known as cardio green, is a polymethine dye with 775 kDa molecular weight, and is a water-soluble anionic photosensitizer. Its negative charge decreases its interaction with negatively charged cell membranes. This photosensitizer has a higher absorbance peak (~800 nm) than the conventional photosensitizers [11]. Unlike other photosensitizers, the primary effect of ICG is due to its photothermal, rather than photochemical effects [12]. Thus, it can more effectively excite the electrons and transfer energy for the generation of free radicals. In fact, due to combined photothermal and photochemical effects, ICG is a suitable agent for effective elimination of endodontic pathogens from hard-to-reach and inaccessible areas. This photosensitizer has a simple application, low cytotoxicity, and is quickly excreted from the body [11].

Laser-activated irrigation (LAI) refers to the activation of irrigants with a specific laser wavelength. Lasers used for this purpose include erbium lasers such as the erbium chromium: yttrium-scandium-gallium-garnet (Er,Cr:YSGG) laser with 2780 nm wavelength, and the erbium: yttrium-aluminum-garnet (Er:YAG) laser with 2940 nm wavelength, which are well absorbed in water, and with their mechanism of action based on causing cavitation in irrigating solutions [13,14]. 

Shockwave-enhanced emission photoacoustic streaming (SWEEPS) is a novel LAI technique suggested for more efficient cleaning of the root canals by using irrigants [15]. In this method, the Er:YAG laser fiber tip is placed in the access cavity filled with irrigant to irradiate the irrigant with paired pulses [16,17]. In this technique, during the collapse of the bubble primarily created by laser irradiation, the second pulse is emitted, creating another bubble, which causes a faster and more violent collapse of the first bubble. The accelerated collapse of the primary bubble, as well as the collapse of the secondary bubble, result in the generation of a shockwave in the irrigant which increases the efficacy of canal disinfection [18]. In other words, the secondary bubble exerts pressure on the primary one and causes its movement into deeper areas and the turbulent movement of the irrigant. For this reason, this method is more efficient than ultrasonic techniques and photon-induced photoacoustic streaming (PIPS) in the elimination of canal debris. In this technique, the determination of the optimal pulse interval is not possible for the clinicians [18]. In auto-SWEEPS mode, which is a more recent technology, this time interval is automatically adjusted between 300–650 µs in 10 µs steps [19]. This study aimed to assess the efficacy of the SWEEPS technique plus aPDT with ICG in eliminating *E. faecalis* biofilm from infected root canals. 

## 2. Materials and Methods 

### 2.1. Sample Preparation 

The study protocol was approved by the Ethics Committee of the Tehran University of Medical Sciences (IR. TUMS. DENTISTRY.REC. 1401. 143). Thirty single-rooted teeth with completely formed roots and mature apices that had been extracted for purposes not related to this study were collected. Immediately after extraction, the teeth were cleaned of tissue residues using a brush, and were stored in saline. Next, the teeth were decoronated at the cementoenamel junction using a high-speed handpiece and diamond fissure bur under air and water spray, such that the root length was standardized to be 12 mm. A #15 K-file (Mani Inc., Tochigi, Japan) was introduced into the canal until its tip was visible at the apex. The working length was determined to be 0.5 mm shorter than this length. The canals were then instrumented with the ProTaper rotary system (Dentsply Maillefer, Ballaigues, Switzerland) up to F4 to the working length with the single length technique, as instructed by the manufacturer. In the process of cleaning and shaping, the root canals were irrigated with NaOCl. In addition, 1 mL of 17% ethylenediaminetetraacetic acid (EDTA) (Masterdent, New York, USA) was used for 3 min for smear layer removal, followed by irrigation with 1 mL of saline, NaOCl, for 3 min, and, as the final irrigation step, the canals were rinsed with 5 mL of sterile saline [20]. The root canals were then dried with #40 paper points. To prevent apical leakage through the apex, the apex of the teeth was sealed with auto-polymerizing glass ionomer (GC Gold Label, Kyoto, Japan). To prevent external microbial contamination, the external root surfaces, except for the canal orifice, were coated with one layer of nail varnish. The teeth were then autoclave-sterilized at 121 °C and 15 Psi pressure for 20 min.

### 2.2. Bacterial Culture

The microorganism used in this study was *E. faecalis* (IBRC-M 11,130), which was obtained from the Iranian Biological Resource Center (Tehran, Iran). *E. faecalis* was cultured in brain heart infusion broth (Ibresco, Iran) under aerobic conditions at 37 °C, overnight. Bacterial suspension with 0.5 McFarland standard concentration (1.5 × 10^8^ colony-forming unit (CFU)/mL) was prepared using a spectrophotometer (optical density (OD) 600 nm: 0.08–0.13). After sterilization of the teeth, the root canals were inoculated with 10 µL of *E. faecalis* bacterial suspension (1.5 × 10^7^ CFU/mL) using a micropipette, and the teeth were incubated at 37 °C for 2 weeks. Ten microliters of fresh microbial suspension were inoculated into the canals every 48 h. After termination of the incubation period, the teeth were rinsed with sterile saline, and randomly assigned to 6 groups. 

### 2.3. Scanning Electron Microscope (SEM) Measurements

After performing the above steps, in order to confirm the formation of biofilm, the teeth were sectioned vertically into two parts and fixed in 1% aqueous osmium tetroxide followed by an ethanol gradient wash, and then sputter coated with gold. The samples were imaged with a SEM-EDAX apparatus (FEI SEM QUANTA 200 EDAX EDS SILICON DRIFT 2017, Hillsborough, OR, USA) at a magnification of 3000×.

### 2.4. Study Groups 

**Figure 1:**
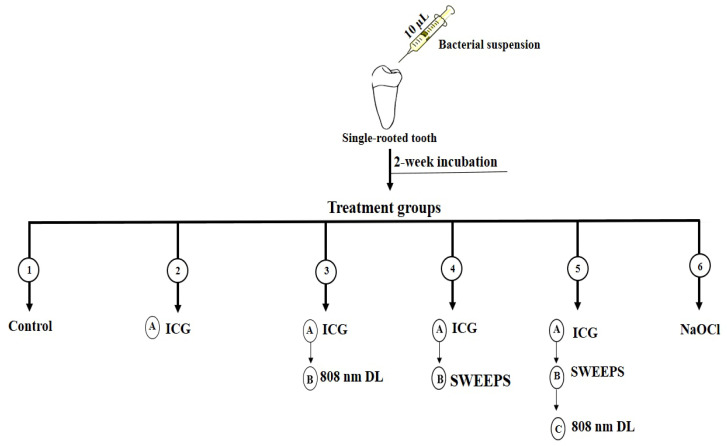
Schematic representation of experimental setup. ICG: Indocyanine green, nm: nanometer, DL: diode laser, SWEEPS: shockwave-enhanced emission photoacoustic streaming, NaOCl: 5.25% sodium hypochlorite.

The treatment steps were as follows (Figure 1):

Group 1. Control group: the teeth did not undergo any intervention.

Group 2. ICG: the root canals were filled with 10 µL of ICG (Green + I, NovaTeb Pars, Tehran, Iran) at a concentration of 1000 µg/mL, and placed at room temperature in the dark for 5 min. 

Group 3. ICG + 808 nm diode laser: the root canals were filled with 10 µL of ICG (1000 µg/mL) and placed at room temperature in the dark for 5 min. They were then subjected to 808 nm diode laser (DX82, Konftec, New Taipei City, Taiwan) with output power of 250 mW and total energy of 15 J, for 60 s. The 3D diffuser tip was used in an up-and-down motion from the apex to the coronal part. 

Group 4. ICG + SWEEPS: the root canals were filled with 10 µL of ICG (1000 µg/mL) and placed at room temperature in the dark for 5 min. They were then subjected to Er:YAG laser irradiation with 2940 nm wavelength (LightWalker AT, Fotona, LjuBlijana, Slovenia) with an H14 handpiece and SWEEPS tip with the Fotona protocol for SWEEPS (25 µs, SWEEPS mode, 15 Hz, 20 mJ, 0.3 W). The tip of SWEEPS was placed in the pulp chamber and activated for 90 s. 

Group 5. ICG + 808 nm diode laser + SWEEPS: the root canals were treated by ICG-mediated SWEEPS similar to group 4, and then, after 5 min, 808-nm diode laser irradiation was performed, similar to group 3.

Group 6. NaOCl: the root canals were filled with 5.25% NaOCl for 1 min. 

### 2.5. Microbiological Process

After treatment, the teeth were placed in a microtube containing 1 mL BHI broth, and vortexed for 1 min. Next, 10 µL of the suspension was serially diluted 5 times, and 10 µL of each dilution was cultured on BHI agar (Ibresco), and incubated at 37 °C for 24 h. The colonies were then counted [11].

### 2.6. Statistical Analysis 

Statistical analysis was carried out using one-way ANOVA (SPSS, version 23.0, Chicago, IL, USA). For multiple comparisons, Tukey’s test was used as the post hoc test. *p* value < 0.05 was considered statistically significant.

## 3. Results 

**Figure 2:**
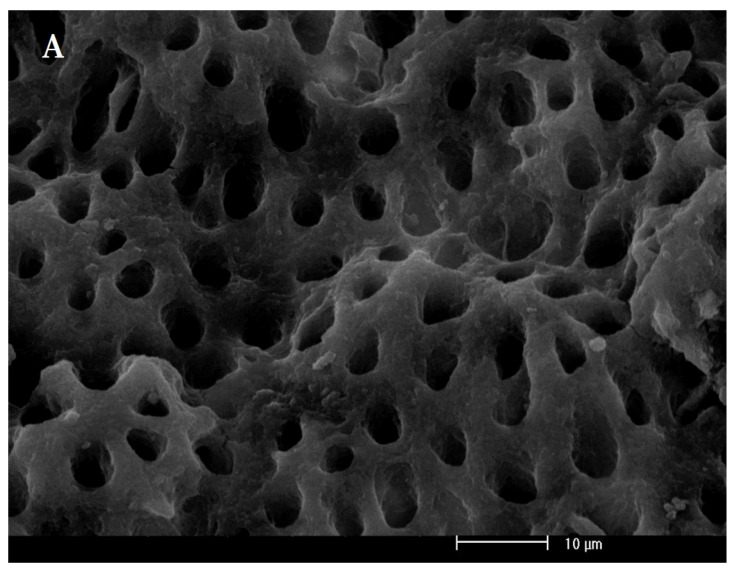

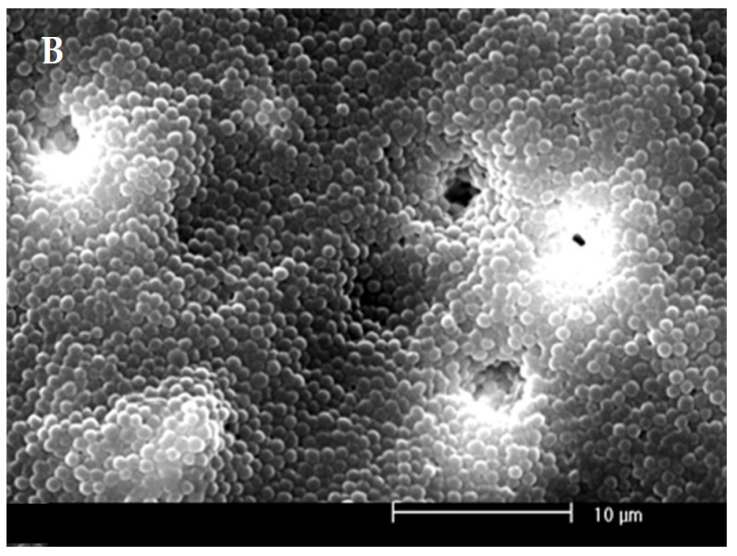
(**A**) Scanning electron microscope of the root canal without bacterial biofilm. (**B**) Scanning electron microscope of *Enterococcus faecalis* biofilm on the root canal walls at a magnification of 3000×.

**Figure 3:**
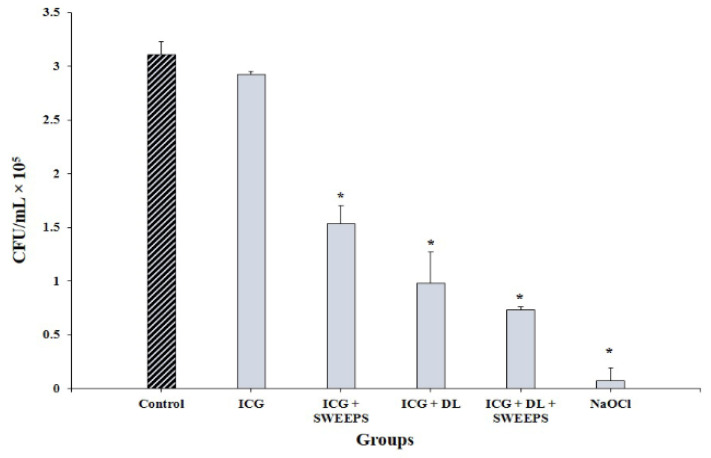
Effect of different treatment groups on cell viability of *Enterococcus faecalis* biofilm. * Significantly different from the control group, *p* < 0.001. ICG: indocyanine green, DL: 808 nm diode laser, SWEEPS: shockwave-enhanced emission photoacoustic streaming, NaOCl: 5.25% sodium hypochlorite.

The SEM of the root canal without bacterial biofilm and *E. faecalis* biofilm on the root canal walls and in the dentinal tubules 2 weeks after inoculation are shown in Figure 2A,B, respectively. The results in Figure 3 and Table 1 demonstrate that, except for ICG alone, all experimental groups could decrease the viability of *E. faecalis*, compared with the control (*p* < 0.001). The results revealed that NaOCl decreased the microbial count to almost zero (*p* < 0.001). A significant difference was found between the ICG and ICG + SWEEPS or 808 nm diode laser, or both (*p* < 0.001) regarding the reduction in the *E. faecalis* count. Accordingly, ICG + 808 nm diode laser + SWEEPS had a 2.1- and 1.3-fold anti-biofilm effect compared to ICG + SWEEPS and ICG + 808 nm diode laser, respectively. In addition, there was a significant difference between the ICG + SWEEPS and ICG + 808 nm diode laser + SWEEPS (*p* = 0.002), whereas there was no significant difference found between the ICG + 808 nm diode laser and ICG + 808 nm diode laser + SWEEPS (*p* = 0.64). Furthermore, ICG + SWEEPS and ICG + 808 nm diode laser groups showed significant differences (*p* = 0.035).

## 4. Discussion 

This study evaluated the efficacy of SWEEPS plus aPDT with ICG for the reduction of *E. faecalis* biofilm from the root canal space. Since studies on the efficacy of the SWEEPS technique in actual root canals are highly limited, this study assessed the efficacy of the above-mentioned techniques in actual root canals with the usual anatomical complexities.

The present results revealed that the application of NaOCl alone had a significantly superior efficacy than the other groups in relation to the reduction of the *E. faecalis* count in the root canal system. For higher disinfecting efficacy, antimicrobial agents need to be in direct contact with the bacteria; however, the lodging of bacteria in anatomical complexities of the canal such as anastomoses, fins, and ramifications make it impossible for the irrigants to directly contact the microorganisms [20]. 

In the present study, aPDT with 808 nm diode laser and ICG caused a significant reduction in the *E faecalis* count, which is in consistent with the results of most studies conducted on the efficacy of aPDT with ICG on *E. faecalis* elimination [21,22]. A study has also shown that there is no evidence of cytotoxicity of ICG to MG-63 human osteoblast-like cells [23]. Furthermore, the results of this study show that ICG + SWEEPS has significant efficacy in removing *E. faecalis* biofilms compared to the control (*p* < 0.001). Wang et al. [24] confirmed the efficiency of the auto-SWEEPS in eliminating *E. faecalis* biofilm in root canals compared to 3% NaOCl alone and PIPS, using SEM images. They explained that strong shockwaves are eventually generated throughout the root canal, which significantly improves clearance efficacy. 

In the SWEEPS technique, a photothermal effect does not occur, due to subablative laser irradiation. This technique generates powerful waves in the irrigating liquid, and produces a high fluid flow rate [25]. The maximum speed of the irrigating solution in accessory canals in the application of the SWEEPS technique is 10 m/s, which is much higher than the reported speed for other methods of root canal irrigation [26]. In addition, the penetration depth of the irrigant into the accessory canals in the SWEEPS technique is more than 1 mm [26]. According to the literature, the optimal efficacy of the SWEEPS technique is attributed to the emission of two pulses with an optimal time interval. Accordingly, the effect of the primary bubbles is reinforced by the generation of secondary bubbles, without increasing the risk of extrusion of the irrigant. Thus, this technique increases the efficacy of irrigation and debris removal from the root canal system [27]. Su et al. [26] described the breath mode for the streaming of irrigants, in which the irrigant repeatedly enters and exits the main and accessory canals. According to their study, another advantage of the SWEEPS technique is that the in-and-out movement of the liquid, which resembles inhalation and exhalation, generates alternating shear stresses in the root canal, which plays a pivotal role in improving the quality of the debridement process. No risk of intracanal instrument fracture is another advantage of the SWEEPS technique, since in this technique the tip of the handpiece is positioned in the access cavity and above the root canals, whereas in sonic and ultrasonic techniques, the instrument is introduced into the canal and proceeds to the apex for the activation of the intracanal irrgant [28]. In addition, a previous study has shown that photosensitizer extrusion is not harmful [29]. 

According to the present results, combination therapy with the application of SWEEPS plus aPDT yielded superior results. The SWEEPS technique mechanically detaches the biofilm from the root canal, therefore increasing the penetration depth and efficacy of the photosensitizer, due to the frequent generation of cavitation [1]. Consistent with the present results, previous studies also showed that application of SWEEPS with a 660 and 980 nm diode laser or light-emitting diode (LED) can cause a more significant decrease in the root canal infected with *E. faecalis* [25,30]. However, in contrast with the results of this study, the diode laser and SWEEPS did not show significant differences with methylene blue in their use as a photosensitizer [30]. Since the type of photosensitizer, light source, and irradiation time affect the bactericidal properties, this difference might be related to the fact that the mechanism of action of ICG is different from that of other photosensitizers. The main effect of ICG is due to the photothermal effect, which causes cell damage by increasing the intracellular temperature [12]. In photothermal therapy, the energy of laser radiation is absorbed by ICG, effectively raising the local temperature [31]. In addition to photothermic effects, ICG was demonstrated to have a photodynamic effect via the production of reactive oxygen species [32]. On the other hand, the 810 nm diode laser compared to other wavelengths used for toluidine blue O and methylene blue allows more penetration depth [12]. The application of only one type of photosensitizer was among the limitations of this study. The in vitro design was another limitation of this study that limits the clinical generalizability of the results. Therefore, future studies are required on other types of photosensitizers and irrigants, and also in the clinical setting.

## 5. Conclusions

The application of ICG along with the SWEEPS plus aPDT significantly improve its efficacy compared with its application alone. The results of this study will probably make an important contribution in the future to improving the efficiency of root canal treatments.

## Figures and Tables

**Table 1:** Multiple comparison post hoc test results.

**Groups**	**Groups**	***p*** **Value**
ICG	Control	0.86
	ICG + DL	<0.001
	ICG + SWEEPS	<0.001
	ICG + DL+ SWEEPS	<0.001
	NaOCl	<0.001
ICG + DL	Control	<0.001
	ICG + SWEEPS	0.035
	ICG + DL+ SWEEPS	0.64
	NaOCl	0.001
ICG + SWEEPS	Control	<0.001
	ICG + DL + SWEEPS	0.002
	NaOCl	<0.001
ICG + DL + SWEEPS	Control	<0.001
	NaOCl	0.01
NaOCl	Control	<0.001

ICG: indocyanine green, DL: 808 nm diode laser, SWEEPS: shockwave-enhanced emission photoacoustic streaming, NaOCl: 5.25% sodium hypochlorite.

## Data Availability

Not applicable.
